# Supplementary material for: The Association of Physical Activity and Sedentary Behavior with Maternal and Cord Blood Anti-Oxidative Capacity and HDL Functionality: Findings of DALI Study
Source: Antioxidants (Basel). 2023 Mar 28;12(4):827. doi: 10.3390/antiox12040827 (PMC10135087; doi:10.3390/antiox12040827)
Supplement: Supplementary file 1 [file antioxidants-12-00827-s001.zip › antioxidants-2269659-supplementary.pdf]

Supplementary Table S1. Characteristics of women selected for analyses and those excluded.

Maternal characteristics	Included N=122	Excluded N=463	P
Age, years, mean ± SD	32.8 ± 5.2	31.6 ± 5.4	0.03
Prepregnancy BMI, kg/m2, mean ± SD	33.7 ± 4.1	33.8 ± 4.3	0.75
Gestational weight gain, kg, mean ± SD	8.2 ± 4.9	7.9 ± 4.7	0.44
Primiparous, n (%)	55 (45%)	242 (52%)	0.16
Married/living with a partner, n (%)	114 (93%)	433 (94%)	0.98
High education, n (%)	80 (66%)	238 (51%)	0.01
European descent, n (%)	108 (89%)	400 (86%)	0.54
Smoking, n (%) n=548	12 (10%)	67 (15%)	0.10
GDM, n (%) n=476	27 (22%)	136 (29%)	0.001
Caesarean Section, n (%) N=516	33 (27%)	134 (29%)	0.17
Spontaneous Delivery, n (%) N=574	74 (61%)	238 (51%)	0.95
Neonatal characteristics			
Gestational age at birth, week, mean ± SD	39.8 ± 1.3	39.5 ± 479.7	0.45
Birthweight, g, mean ± SD	3592 ± 480	3439.5 ± 570.3	0.01
Female sex, n (%) n=529	57 (47%)	201 (43%)	0.61

Supplementary Table S2. Associations (linear regression models) of maternal moderate-to-vigorous physical activity (MVPA) and sedentary time (ST) with pro/anti-oxidative stress and HDL-related markers in cord blood.

< 20 weeks (n=44)	AOPP[a], umol/L B (95% CI)	P	Inhibition of oxidation, % B (95% CI)	P	AE activity, mM/min/ml B (95% CI)	P	Cholesterol Efflux, % B (95% CI)	P
Sedentary time (% weartime)	0.01 (-0.02, 0.03)	0.09	**0.38 (0.18; 0.59)**	**<0.001**	0.11 (-0.99; 1.23)	0.84	0.01 (-0.06; 0.08)	0.73
MVPA (min/day)	0.01 (-0.03, 0.01)	0.20	**0.13 (0.05; 0.22)**	**0.003**	**0.59 (0.14; 1.05)**	**0.01**	-0.01 (-0.04; 0.02)	0.46
**24-28 weeks (n=38)**								
Sedentary time (% weartime)	0.01 (-0.01, 0.03)	0.27	0.20 (-0.03; 0.43)	0.08	0.16 (-0.98; 1.29)	0.78	-0.01 (-0.08; 0.06)	0.90
MVPA (min/day)	0.003 (-0.01, 0.01)	0.5	**0.11 (0.02; 0.21)**	**0.02**	**0.49 (0.06; 0.98)**	**0.05**	-0.00 (-0.03; 0.03)	0.81
**35-37 weeks (n=34)**								
Sedentary time (% weartime)	0.02 (-0.002, 0.03)	0.07	0.2 (-0.09; 0.49)	0.16	-0.70 (-1.67; 0.27)	0.15	-0.03 (-0.11; 0.06)	0.54
MVPA (min/day)	**0.01 (0.003, 0.02)**	**0.01**	**0.16 (0.02; 0.31)**	**0.03**	0.32 (-0.16; 0.8)	0.18	0.00 (-0.04; 0.04)	0.98

[a] log transformed values were used in the regression analyses. All models adjusted for maternal BMI, age and parity. MVPA and sedentary time are simultaneously in the models. Thus, they are adjusted for /independent of each other. Bold font indicates significant associations. B=Beta, and CI= Confidence Interval.

Supplementary Table S3. Associations (linear regression models) of maternal moderate-to-vigorous physical activity (MVPA) and sedentary time (ST) with pro/anti-oxidative stress and HDL-related markers in cord blood.

**< 20 weeks (n=37)**	**AOPP[a], umol/L B (95% CI)**	**P**	**Inhibition of oxidation, % B (95% CI)**	**P**	**AE activity, mM/min/ml B (95% CI)**	**P**	**Cholesterol Efflux, % B (95% CI)**	**P**
Sedentary time (% weartime)	0.01 (-0.01; 0.01)	0.46	**0.32 (0.10; 0.54)**	**0.01**	-0.028 (-1.47; 0.91)	0.63	0.01 (-0.06; 0.08)	0.71
MVPA (min/day)	0.01 (-0.01; 0.03)	0.21	**0.11 (0.01; 0.21)**	**0.03**	0.49 (-0.05; 1.03)	0.08	-0.01 (-0.05; 0.02)	0.36
**24-28 weeks (n=35)**								
Sedentary time (% weartime)	0.01 (-0.01; 0.03)	0.23	0.16 (-0.06; 0.39)	0.14	-0.003 (-1.15; 1.14)	0.99	-0.001 (-0.08; 0.07)	0.97
MVPA (min/day)	0.003 (-0.00; 0.01)	0.36	**0.12 (0.03; 0.22)**	**0.01**	0.47 (-0.01, 0.95)	0.06	-0.001 (-0.03; 0.03)	0.95
**35-37 weeks (n=31)**								
Sedentary time (% weartime)	0.02 (-0.004; 0.04)	0.12	0.17 (-0.13; 0.46)	0.25	-0.63 (-1.56; 0.31)	0.18	-0.04 (-0.14; 0.05)	0.36
MVPA (min/day)	**0.01 (0.003; 0.02)**	**0.01**	**0.18 (0.04; 0.32)**	**0.02**	0.42 (-0.03; 0.87)	0.07	-0.004 (-0.05; 0.04)	0.85

[a] log transformed values were used in the regression analyses. All models adjusted for maternal BMI, age and parity. MVPA and sedentary time are simultaneously in the models, Thus, they are adjusted for /independent of each other. Bold font indicates significant associations. B=Beta, and CI= Confidence Interval.
